# Three-Dimensional Scaffolds for Bone Tissue Engineering

**DOI:** 10.3390/bioengineering10070759

**Published:** 2023-06-25

**Authors:** Harish Chinnasami, Mohan Kumar Dey, Ram Devireddy

**Affiliations:** Department of Mechanical Engineering, Louisiana State University, Baton Rouge, LA 70803, USA; cshmech@gmail.com (H.C.);

**Keywords:** tissue engineering, bone grafts, autograft, allograft, xenograft, calcium phosphates, human mesenchymal stem cells (hMSCs), osteoblasts, porosity, compressive strength/modulus, 3D bioprinting, freezing, mechanical properties, regulatory issues

## Abstract

Immobilization using external or internal splints is a standard and effective procedure to treat minor skeletal fractures. In the case of major skeletal defects caused by extreme trauma, infectious diseases or tumors, the surgical implantation of a bone graft from external sources is required for a complete cure. Practical disadvantages, such as the risk of immune rejection and infection at the implant site, are high in xenografts and allografts. Currently, an autograft from the iliac crest of a patient is considered the “gold standard” method for treating large-scale skeletal defects. However, this method is not an ideal solution due to its limited availability and significant reports of morbidity in the harvest site (30%) as well as the implanted site (5–35%). Tissue-engineered bone grafts aim to create a mechanically strong, biologically viable and degradable bone graft by combining a three-dimensional porous scaffold with osteoblast or progenitor cells. The materials used for such tissue-engineered bone grafts can be broadly divided into ceramic materials (calcium phosphates) and biocompatible/bioactive synthetic polymers. This review summarizes the types of materials used to make scaffolds for cryo-preservable tissue-engineered bone grafts as well as the distinct methods adopted to create the scaffolds, including traditional scaffold fabrication methods (solvent-casting, gas-foaming, electrospinning, thermally induced phase separation) and more recent fabrication methods (fused deposition molding, stereolithography, selective laser sintering, Inkjet 3D printing, laser-assisted bioprinting and 3D bioprinting). This is followed by a short summation of the current osteochondrogenic models along with the required scaffold mechanical properties for in vivo applications. We then present a few results of the effects of freezing and thawing on the structural and mechanical integrity of PLLA scaffolds prepared by the thermally induced phase separation method and conclude this review article by summarizing the current regulatory requirements for tissue-engineered products.

## 1. Introduction

A remarkable phenomenon of the human skeletal system is its restorative healing capacity with immobilization [1]. However, incomplete healing can occur in specific sites of injuries at lower limb joints. This leads to a condition called pseudoarthrosis, where complete bone fusion to its original form is impossible without the surgical implantation of external grafts [2]. Three-dimensional scaffolds are used to make tissue-engineered bone grafts for treating traumatic defects by surgical implantation [3] and by controlled drug delivery [4]. In rare cases, bone grafting has been used to treat tumors by curettage and cementation methods [5]. Scaffolds used in making such implantable grafts should exhibit micro- and macrostructures that mimic the recipient’s host tissue. Moreover, the scaffold material should be biocompatible and nontoxic and facilitate in vivo integration after implantation. Note that the microstructure (pore size and porosity) of the scaffolds plays a crucial role in the cellular growth and differentiation of progenitor cells (stem cells) [6]. A viable bone graft should, thus, possess the following integral properties: *osteoinductive*: induces the differentiation of osteoprogenitor cells (stem cells) into osteogenic lineages; *osteoconductive*: favorably influences the proliferation of osteoblasts and blood vessel incursion, leading to the formation of an osteoid; and *osteogenic*: the implanted graft material should itself integrate with the host bone, simultaneously maintaining the viability of cells.

The first described bone grafting was performed in 1668. A cranial defect of an injured soldier was repaired using a dog’s skull [7]. An ever-increasing demand for bone grafting has followed since then (due to trauma, tumor excision, spinal fusion, etc.) calling for expanded research for skeletal reconstructions. Musculoskeletal defects restricted the lives of over 1.5 million Americans in 2015, and the numbers have only increased during the intervening years [8]. About 2.2 million bone grafting procedures are performed worldwide annually [9]. Many of these disorders require surgery to accelerate or improve bone repair. One common example is spinal fusion surgery, which is recommended to treat back pain secondary to scoliosis, spinal stenosis, degenerative disc disease, infectious processes, tumors and trauma. At present, the “gold standard” for spinal fusion repair is autologous bone, which is usually harvested from the iliac crest of the individual [10]. Nevertheless, this is far from a perfect solution.

Autografts (previously known as homografts) are the most osteogenic material containing live osteocytes, increasing the chances of fusion. Although autografting may seem like a convenient ideal method for bone grafting, it is limited by numerous practical disadvantages. These include a high chance of morbidity in the donor (iliac crest, rib, fibula, etc.) as well as at the host (injured) sites. Rates of pseudoarthrosis also range from 5% to 35% in the healed site. These disadvantages are only further complicated by low availability, risk of blood loss and transfusion, increased surgical time and cost [11]. In 30% of patients, the donor site becomes infected, bruised, fractured or painful following the surgery [12]. Indeed, when a patient requires bone autografts for multiple spinal fusions, the iliac crest may not provide sufficient material [13]. While there are alternative materials available, all face a common limitation: a lack of osteogenic capability. Allograft bone from cadavers can be sterilized, stored and used in the operating room as needed. These materials can be pre-shaped for specific use or powdered, allowing them to be applied as a paste at the surgical site [14]; however, allografts can cause inflammation and elicit an immune response and have been an infectious source in a limited number of cases [15]. Because allograft bone is sterilized, it no longer contains viable native bone forming cells (osteoblasts, osteocytes) and lacks osteogenic properties. In clinical trials, allograft bone is inferior to autograft bone in multilevel spinal fusions [16].

**Figure 1:**
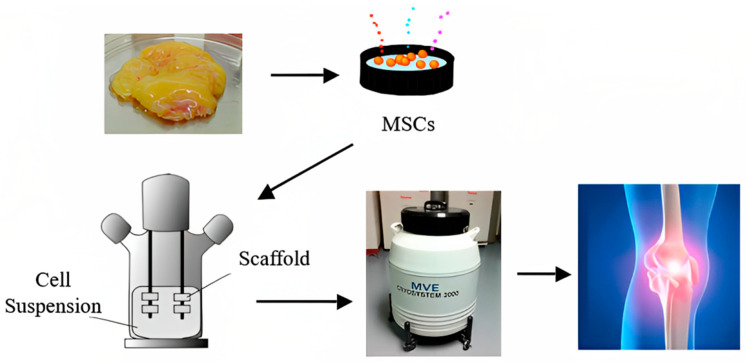
A futuristic paradigm for bone tissue engineering.

A group in the National University of Singapore collaborating with Temasek Polytechnic has developed, calibrated and patented a novel PCL-ceramic (HA/TCP) using the fused deposition modeling (FDM) technique. These FDM-fabricated implants are being used as burr hole plugs in cranioplasty and to regenerate the iliac crest after an autograft was taken. The clinical outcomes were positive, with no patients developing adverse side-effects a year after surgery [17]. Therefore, it is extremely important to have a better understanding of the various factors involved in tissue engineering a viable bone graft. This review reports on some commonly used materials and methods intended to make bone grafts and their in vivo outcomes on some mammalian species. Future adaptations of such tissue-engineered grafts are expected to be significantly enhanced and facilitated with appropriate cryobiological studies; a paradigm for such a future use of cryopreserved tissue-engineered grafts is shown in Figure 1.

## 2. Scaffold Materials: Ceramics and Polymers

Ceramics such as tri-calcium phosphates (TCP) (Ca_3_(PO_4_)_2_) have been used in bone repair for the past 80 years [18]. The stoichiometry of TCP is similar to an amorphous precursor to the inorganic composition of bone. A significant advantage of α- and β-tricalcium phosphates is their solubility in water, enhancing in vivo degradation [18]. α- and β-tricalcium phosphates have similar chemical compositions; however, they have different crystallographic structures, making αTCP more soluble in water. αTCP is obtained by heating βTCP at high temperatures (1150 °C) followed by quenching [19]. The most common bone scaffolds made from βTCP have a porosity ranging from 35–50% and pore sizes ranging from 100–300 µm [20].

Hydroxyapatite (HA) (Ca_10_(PO_4_)_6_(OH)_2_—crystal unit) is another commonly used calcium orthophosphate compound [18]. It is preferred due to its stoichiometric similarity to the minerals naturally present in bones. A variety of bone substitutes made of HA, such as Pro Osteon 500R (Interpore Cross International, Irvine, CA, USA), Bio-Oss^®^ (Geistlich Pharma North America Inc., Princeton, NJ, USA) and Endobon^®^ (Zimmer Biomet, Palm Beach Gardens, FL, USA), are available commercially [21]. Pro Osteoan 500R is made by demineralizing natural coral exoskeletons and hydrothermal conversion of calcium carbonate into calcium phosphate. Incomplete conversions result in external HA and internal calcium carbonate, which are more resorbable [22]. The resorption rates of HA in vivo are very low at 5–15% per year [20].

HA is comparatively more crystalline than βTCP. Hence, scaffolds made from HA have higher mechanical strength comparable to cancellous bone. However, βTCP promotes in vivo degradation. To combine the advantages of these two compounds, composites of βTCP and HA (biphasic calcium phosphates) are used as scaffold materials. The resorption of the implants depends on the composition of βTCP: HA. The most common compositions of either of these compounds are 40–60%. Some commercially available bone substitutes are Triosite (Zimmer Biomet, Palm Beach Gardens, FL, USA) and BCP bone void filler (Medtronic Sofamor Danek USA, Inc., Memphis, TN, USA) [18].

Basically, the sintering process or with minor modifications is used to make pure ceramic scaffolds. The microstructural property in such methods is partially controlled by using a negative mold made of industrial foam such as polyurethane. This mold will be removed during the heating process. A combination of gel casting and a polymer sponge mold was used by Ramay et al. to make porous HA scaffolds by sintering at 1350 °C for 2 h [23]. Alternatively, the phase separation method was adopted by Fukasawa et al. by freeze-drying water-based ceramic slurry [24]. In general, the pore sizes of such scaffolds were in the order of few hundred µm to few millimeters, but the porosity was as low as 30–40%. This, combined with the low biodegradability of materials such as HA, led scientists to study biodegradable polymers for bone grafts.

Polymers can be divided into two major categories: naturally available polymers such as polysaccharides (starch, alginate, chitin/chitosan, hyaluronic acid derivatives) or proteins (soy, collagen, fibrin gels, silk) [25] and synthetic biodegradable polymers (PLA, PGA and PCL). This review article will focus on synthetic biodegradable polymers due to their compatibility with bone graft synthesis. Scaffolds made using synthetic polymers can be produced under controlled conditions with predictable and reproducible mechanical (compressive/tensile strength and modulus) and structural properties (pore-sizes, pore shapes and porosity (%)). Moreover, using synthetic polymers averts the risks of material impurities and toxins in the bone graft, which lead to immune rejections in the host.

**Figure 2:**
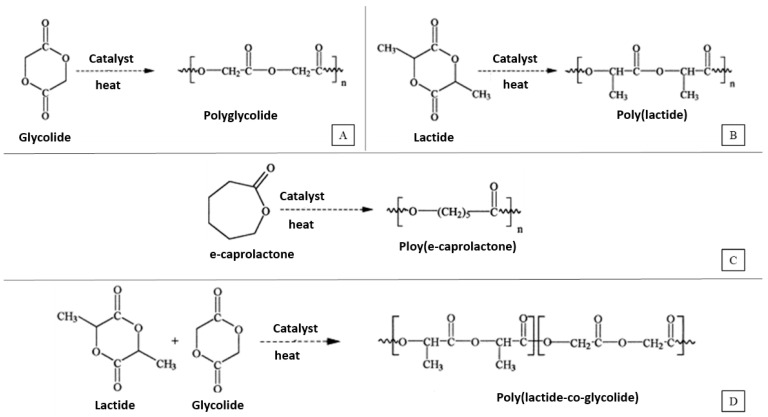
Synthesis of (**A**) Poly(glycolic acid) (PGA), (**B**) Poly(l-lactic acid) (PLLA), (**C**) Poly(ɛ-caprolactone) (PCL) and (**D**) Poly(lactic-co-glycolic acid) (PLGA).

Some commonly used synthetic polymers used to make tridimensional scaffolds for bone tissue engineering are saturated poly-α-hydroxy esters, namely poly(lactic acid) (PLA) and poly(glycolic acid) (PGA), as well as poly(lactic-co-glycolide) (PLGA) copolymers [26]. PLA exists in three forms: l-PLA (PLLA), d-PLA (PDLA) and a racemic mixture of d,l-PLA (PDLLA). Figure 2, adapted from [27], shows the chemical structure and synthesis of PGA, PLLA, PCL and PLGA.

Poly(ε-caprolactone) (PCL), an important member of the aliphatic polyester family, has been used to entrap antibiotic drugs. Thus, a composite of PCL and antibiotic drugs can be considered as an effective drug-delivery system [28]. These composites can be used in the treatment of bone defects by enhancing bone ingrowth and regeneration. The degradation mechanism of PCL is similar to that of PLA undergoing a hydrolytic degradation of ester bonds. However, the degradation time for PCL is higher compared to other polymers, taking up to several years in some cases [29].

The degradation times given in Table 1, adapted from [30], represent a collection of the observed data so far in the published literature. However, the actual degradation times of the grafts in vivo would depend on a variety of factors, such as pore morphology, porosity (%), mechanical strength, crystallinity, molar mass (Mw) and polydispersity (Mw/Mn). In general, the degradation rates are as follows [30]: PGA > PDLLA > PLLA > PCL. When using PLA and PGA or their other forms (PLLA, PDLA, PLGA) as scaffold materials for bone tissue engineering, there is always a risk of bulk degradation causing the implant to fail pre-maturely. This quick degradation causes an influx of acidic by products, which may lead to inflammatory responses in the host tissue [31].

More recently, bioactive ceramics have been utilized due to their ability to stimulate the differentiation of stem cells to osteoblasts, and they belong to the family of osteoconductive materials. The most used bioactive ceramic materials are calcium phosphate CaP and silicate-based ceramics. These materials also have an affinity to biointegrate with the host tissue and alleviate bone defects [32]. Comesaña et al. presented an interesting application of rapid prototyping based on laser cladding for the fabrication of composition-gradient bioceramic implants. The concept of having a CaP inner core and a bioactive glass outer layer with varying degradation rates is innovative and could potentially address the need for controlled resorption implants in craniofacial reconstructive surgery [33].

## 3. Scaffold Fabrication Methods

*Solvent-Casting and Particulate Leaching Technique:* In this method of fabricating scaffolds, water-soluble salt (e.g., sodium chloride, sodium citrate) particles are mixed into a biodegradable polymer solution. The solvent is then removed by lyophilization. The salt particles are leached out to obtain a porous structure. The advantages of this method are its simple operation and the ability to the control pore size and porosity by varying the salt/polymer ratio and particle size of the added salt [34].

**Figure 3:**
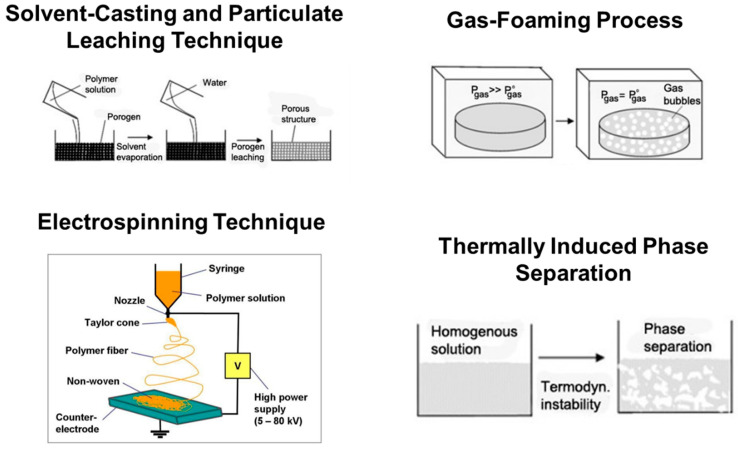
Schematic illustrations of basic scaffold fabrication techniques. (Adapted from Ref. [38]).

*Gas-Foaming Process:* In this method, solid polymer disks are saturated with highly pressurized CO_2_. Releasing CO_2_ gas from the polymer system causes thermodynamic instability (as shown in Figure 3). This makes CO_2_ bubbles grow inside the composite, forming a 3D porous polymer structure. With this technique, scaffolds with ~93% porosity and a pore size of ~100μm can be fabricated. No organic solvents are used in this method. The disadvantage of this method is that interconnectivity of pores is too low [35].

*Electrospinning Technique:* A polymer solution or melt is induced with an electric potential (as shown in Figure 3). At a critical voltage, the charge imbalance overcomes the surface tension, forming a polymer jet. This jet is directed onto a substrate as the solvent evaporates and polymer fibers are formed. Localized control on the microstructure of the porous scaffold can be achieved. The only disadvantage of this method is that it is very tedious to make a 3D scaffold of considerable thickness. Scaffolds made by this process are usually sheets with thickness in the range of microns [36].

*Thermally Induced Phase Separation (TIPS):* This is a simple procedure in which the polymer is first dissolved in a solvent at a higher temperature (as shown in Figure 3). Liquid–liquid or solid–liquid phase separation is induced by lowering the temperature until the composite is frozen. In most cases, low temperatures are reached by using a controlled supply of liquid nitrogen vapors (−160 °C). Hence, thermoplastic synthetic polymers are more suitable for this method of scaffold fabrication due to their stability at low temperatures compared to inorganic ceramics or organically available hydrocarbons. The frozen solvent is sublimated by maintaining low pressure and temperature, leaving a porous polymer scaffold. The pore morphology of the scaffolds varies depending on the concentration of the polymer solution and induced thermal profile during the freezing process. Usually, scaffolds fabricated by this method have good mechanical properties. Ma et al., 2001 [37] observed that PLLA scaffolds fabricated using this technique have a compression modulus about 20 times higher than that of scaffolds fabricated using other methods. This method usually generates scaffolds with a pore size of 30–150 µm [37].

*Solid Freeform Fabrication (SFF):* Computer-aided design (CAD) models are used to tridimensionally print a polymer scaffold of predesigned micro and macro structures. One early form of SFF known as rapid prototyping technique (RPT) was first developed and published by Bredt et al., 1990 at the Massachusetts Institute of Technology, Cambridge, MA, USA [39]. A polymer jet is targeted on a substrate similar to Inkjet printing, forming complex 3D scaffolds of sequential layers. Low molecular weight PLLA was used to print scaffolds with a tensile strength of up to 17 MPa [40]. However, the practical disadvantage of this method is the thick layers of scaffold material, which are determined by the thickness of the polymer jet. This essentially decreases the overall porosity (%) of the scaffold. Coupling the SFF method with other conventional scaffold fabrication processes (solvent-casting, phase separation, gas-foaming) could result in an ideal scaffold with distinguishable micro- and macroporous structures while at the same time increasing the porosity. In 2002, Xiong et al. from Tsinghua University, Beijing, China, developed such a combination method. PLLA/TCP/dioxane composites were printed using the low-temperature deposition method (LDM). With this method, the measured porosity values were about 90% [41]. These PLLA/TCP composite scaffolds loaded with bovine bone morphogenic protein (bBMP) were implanted to repair 20 mm segmental defects in canine radiuses. The defects were found to be completely cured 24 weeks after implantation. The long fabrication time and high manufacturing costs are critical limiting factors for using this technique. This is the reason why the majority of in vitro and clinical studies for bone grafting still use conventional scaffold fabrication techniques.

## 4. 3D Printing Fabrication Method

**Figure 4:**
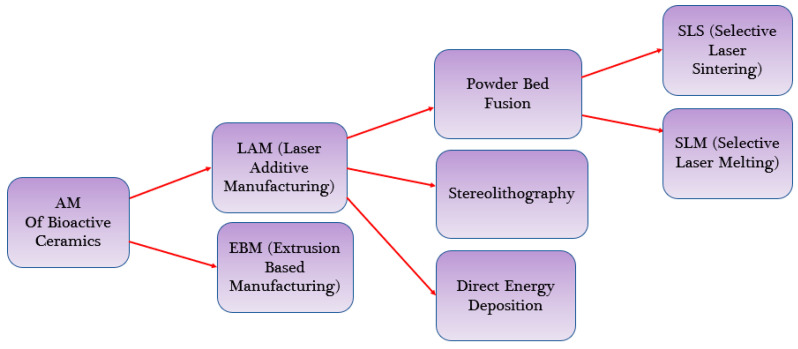
AM methods Used for Bioactive Ceramics.

Additive manufacturing (AM), or 3D printing, allows for the creation of complex, interconnected porous structures that mimic the natural architecture of tissues. Numerous AM methods have been utilized to produce scaffolds with bioactive ceramics, including L-PBF, material extrusion (ME), stereolithography (SLA) [42], directed energy deposition (DED), binder jetting (3DP) and vat polymerization, as shown in Figure 4 [32]. L-PBF, also known as laser additive manufacturing through the powder bed or laser additive manufacturing (LAM), combines two techniques (SLS and SLM) and is collectively referred to as PBSLP. PBSLP is a highly attractive method used for functionalizing biomaterials, enabling the development of personalized medicine and drug delivery systems. One of its unique capabilities is the direct incorporation of temperature-sensitive biomolecules, such as drugs, into the powder bed [43]. This can be achieved either during scaffold fabrication or by adjusting the SLM or SLS parameters [44]. PBSLP offers the flexibility to print biomaterials with tailored engineering properties, including immediate release, control over drug release, multilayered structures and visually impaired print lets. This technology holds great potential for personalized medicine by targeting specific patient groups [45].

**Figure 5:**
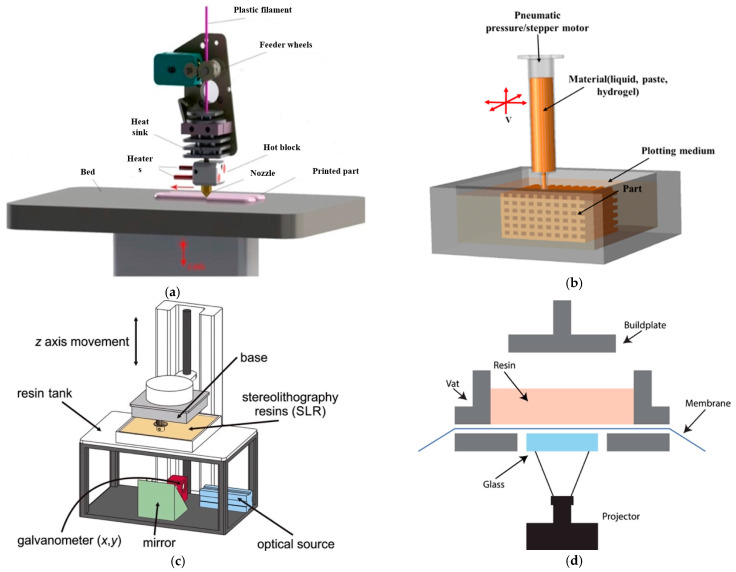

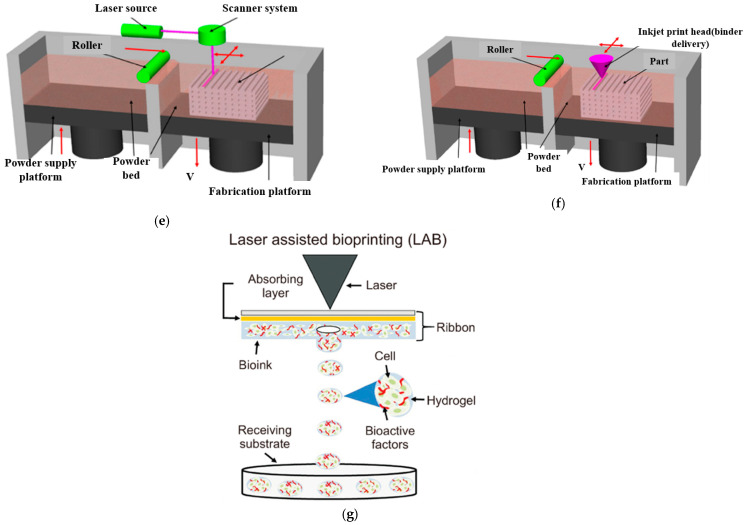
A compilation/schematic of various 3D printing processes. (**a**) FDM [58], (**b**) 3D Bioprinter/Plotting [57], (**c**) SLA [59], (**d**) DLP [60], (**e**) SLS [57], (**f**) 3DP [57], (**g**) LAB [52].

The 3D printing method uses computer-aided design (CAD) software to create a 3D model of the scaffold and then prints it using a variety of materials, including polymers and ceramics. Three-dimensional printing works by laying down successive layers of material until the entire object is created. There are several types of 3D printing technologies that have been used for bone tissue engineering, such as fused deposition modeling (FDM) [46], two types of stereolithography (SLA) [47] (laser-based stereolithography [48] and digital light processing-based stereolithography (DLP)) [49], selective laser sintering (SLS) [50], Inkjet 3D printing (3DP) [51], laser-assisted bioprinting (LAB) [52] and 3D bioprinting [53]. The schematic diagram of these technologies is shown in Figure 5, and the working principle and the pros and cons of these technologies are described in Table 2. The FDM technologies have the advantage of being cost-effective and easy to use, but they lack precision in the vertical direction. On the other hand, SLS and LAB have high waste production but have better resolution in the vertical axis [54]. Another method is layer-wise slurry deposition, which is a 3D printing technique that involves the sequential deposition of layers of a slurry material to create three-dimensional objects [55]. This technique is also known as layer-wise slurry printing or slurry-based 3D printing. In layer-wise slurry deposition, the slurry is typically composed of a powdered material suspended in a liquid binder. The powdered materials can be metals, ceramics, polymers or composites, depending on the desired properties of the final object. The liquid binder acts as a temporary adhesive, holding the powdered particles together during the printing process. The printing process starts with a digital model of the object that needs to be printed. This model is sliced into thin cross-sectional layers, and each layer is then printed one by one. The slurry is spread onto a build platform, and a print head or nozzle deposits the slurry layer by layer according to the sliced model. Once a layer is deposited, it is often subjected to some form of post-processing. This can involve methods such as drying, curing, or sintering to solidify or strengthen the layer before the next layer is added. The process is repeated until the entire object is printed. Layer-wise slurry deposition offers several advantages over other 3D printing techniques. It allows for the creation of complex shapes and intricate internal structures that are difficult or impossible to produce with traditional manufacturing methods. It also enables the use of a wide range of materials, including those with specific properties such as high strength, heat resistance, or electrical conductivity. However, it is worth noting that layer-wise slurry deposition also has some limitations. The process can be relatively slow compared to other 3D printing methods, as each layer requires post-processing before the next layer can be added. Researchers have recently been focusing on developing materials that can be cured by light and are more biocompatible for use in SLA and DLP, as many current light-curable polymers are dissolved in organic solvents that are not biocompatible [56]. The limitations of 3D printing material have broadly been studied by many researchers, as reviewed elsewhere [57].

## 5. Bioactive Ceramics

Ceramics have played a crucial role in the advancement of bone tissue engineering inspired by nature. Specifically, ceramics based on calcium phosphate (CaP) closely resemble the inorganic part of the bone matrix in terms of structure and chemical properties. These ceramics are typically formed into porous scaffolds that mimic the native bone matrix. The CaP ceramics consist of two important phases: hydroxyapatite (HA) and beta-tricalcium phosphate (β-TCP). HA is known for its ability to substitute various ions in its lattice structure and has an advantage in bone regeneration due to its piezoelectric behavior, which helps accelerate the bone healing process [69]. Ahlhelm et al. [70] used a combination of additive manufacturing (CerAM VPP) and freeze foaming to create mechanically stable tricalcium phosphate (TCP) structural hybrid scaffolds for designing a prospective bone replacement. When compared to a bone grafting Curasan material as a standard, this bone construct had a 25-fold higher compressive strength than the pure CaP freeze foam and great biocompatibility with human osteoblastic MG-63 cells. Liu et al. [71] demonstrated the feasibility of creating HA and nano-silica sol composite scaffolds using selective laser sintering (SLS) with a custom-made printer. They also successfully fabricated mechanically robust β-TCP and 45S5 bioglass scaffolds. By optimizing the heating and cooling processes during SLS, they transformed the composition of the bioglass scaffolds into a favorable crystallization phase called Na_2_Ca_2_Si_3_O_9_. Heat treatment at 1200 °C was applied to enhance the mechanical stability by binding the SLS layers. The resulting scaffolds had pore sizes ranging from 750 to 1050 µm. Surface roughness analysis using atomic force microscopy (AFM) showed that SLS was effective in producing rough scaffolds with a roughness of 525 nm, which facilitated the attachment of bone marrow-derived osteoprogenitor cells [72].

However, processing CaP bioceramics through powder bed selective laser processing (PBSLP) presents challenges. The laser’s impact on the powder bed can lead to excessive grain growth, phase development and decomposition at temperatures of 1250–1300 °C [73]. Undesirable phases such as calcium oxide, alpha-tricalcium phosphate (α-TCP) and tetra calcium phosphate (TTCP) may form, potentially affecting the mechanical properties of the CaP scaffolds. In a study by Shuai et al., [74] the potential of a CO_2_ laser operating at a wavelength of 10.6 μm to sinter the HA powder bed was investigated. The results showed that by varying the energy density from 2 to 4 J/mm^2^, no additional phases other than HA were formed. The scaffold produced at 4 J/mm^2^ exhibited the highest Vickers hardness of 4 GPa and a fracture toughness of 1.28 MPa·m1/2, making it suitable for cancellous bone [32]. Several studies have also explored the use of metal matrices such as stainless steel, pure titanium and Ti_6_Al_4_V (Ti64) along with CaP fillers to enhance osseointegration through direct powder bed selective laser processing, particularly for load-bearing applications [75,76]. However, incorporating a metal component in the HA composite can lead to the phase transformation of HA due to the metal’s higher melting temperature. This challenge can be overcome by introducing a polymer matrix along with CaP fillers in the direct powder bed selective laser processing method, allowing the fabrication of polymer-bioceramic bioactive scaffolds [77,78]. Table 3 presents a compilation of studies conducted by various researchers that have investigated the utilization of HA reinforced composites in the field of bio tissue engineering.

## 6. Present Status of 3D Printing Technology for Bone Fabrication

The current state of 3D printing technology for bone fabrication is rapidly advancing. Various laboratory animals such as rats, mice, rabbits, dogs, sheep, goats and pigs have been used as animal models to study bone graft substitutes due to their bones’ similarities to human bones. A recent study by Zhang et al. [88] investigated the potential of electrohydrodynamic 3D printing technology to create scaffolds for Achilles tendon (AT) defects using poly-(ɛ-ɛ-caprolactone) (PCL) and pluronic F127 (F127). The authors found that the inclusion of F127 in the scaffold improved the hydrophilicity and degradation of PCL in vitro. PCL scaffolds with 5% F127 were identified as the most suitable for cell adhesion and growth, indicating good biocompatibility in vitro. The researchers then transplanted these scaffolds with C3H10T1/2 cells into rat models with AT defects. Histological analysis of the transplanted scaffolds revealed that they were beneficial for the accumulation and arrangement of collagen fibers, providing a promising avenue for tissue engineering scaffolds for ATs. Boccaccini et al. [89] used pre-osteoblast MC3T3E1 cells to demonstrate the printability of PLA-BG filaments as well as the bioactivity and cytocompatibility of PLA-BG scaffolds. Gene expression analysis showed that BG inclusions in FDM scaffolds had a positive effect on osteoinduction because they improved the osteogenic differentiation of human-adipose-derived stem cells compared to pristine PLA. Their results demonstrated that FDM is a practical additive manufacturing technology for creating PLA-BG composite scaffolds suited for bone tissue engineering. Nandi et al. [90] suggested valuable insights into the use of 3D-printed SiO_2_ and ZnO-doped tricalcium phosphate (TCP) scaffolds for bone regeneration and healing. The authors used a well-established rabbit tibial defect model to investigate the effects of these scaffolds on in vivo bone formation. The findings of this study demonstrate that the addition of dopants to the TCP scaffolds significantly improved their osteogenic capabilities and accelerated bone formation and healing. Kim et al. [91] studied in vitro biocompatibility evaluations using osteoblast-like cells (MG63), and the assessment of alkaline phosphatase (ALP) activity and calcium deposition provided valuable information on the effectiveness of the composites in promoting bone tissue growth. The results suggest that PCL/bone formation peptide (BFP-1)/alginate has significantly higher ALP activity and calcium deposition than the PCL/bone morphogenic protein (BMP-2)/alginate composite. This finding is important, as it suggests that BFP-1 can be a good growth factor for enhancing bone tissue growth, and the simple-alginate coating method is a useful tool for fabrication of highly functional biomaterials through release–control supplementation.

**Figure 6:**
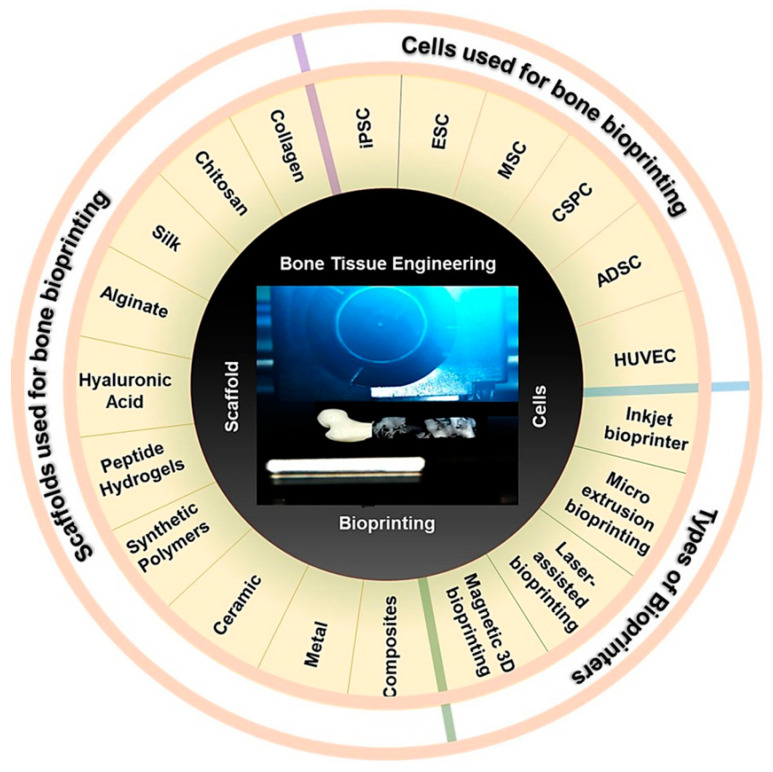
3D printing of bone. Various cell types, scaffold materials and bioprinting technology being used for 3D printing bone and bone scaffolds [93].

Bendtsen et al. [92] developed a novel alginate-PVA-hydroxyapatite (HA) hydrogel formulation with optimal rheological properties for 3D bioprinting of mouse calvaria 3T3-E1 (MC3T3) cells into scaffolds of high shape fidelity. The incorporation of HA in the hydrogel formulation provides a favorable bone-forming environment due to its excellent osteoconductivity. The systematic investigation of varying concentrations of alginate, phosphate, calcium and PVA-HA suspension in the formulation on the resulting viscosity and thus printability of the hydrogel is an important aspect of this study. The degradation studies in α-MEM cell culture media demonstrated that the 3D-printed alginate-PVA-HA scaffolds remained intact for 14 days, which is a promising result. Bisht et al. [93] have provided a substantial overview of the present state of 3D printing technology for bone manufacturing, which is encapsulated in Figure 6. Recent breakthroughs in material science, imaging technology and software design have enabled the creation of 3D-printed bone implants that are custom-fit and biocompatible and have the potential to integrate more seamlessly with existing bone tissue. Montero et al. [94] investigated the formulations of NaAlg/PNIPAm/ZnSO_4_-based smart bioinks and their characterization through various analytical techniques, including SEM, rheological analysis and hemolysis assay, is impressive. The finding that the presence of the Zn^2+^ ion and the extrusion process affect the morphology of the scaffolds is noteworthy. These 3D-printed implants are being used for a wide range of applications, including reconstructive surgery, dental implants and orthopedic implants. However, there are still challenges that need to be addressed, such as the need for more long-term studies to evaluate the safety and efficacy of 3D-printed bone implants. Nonetheless, the progress made in this field holds great promise for the future of regenerative medicine and personalized healthcare.

## 7. Printing Scaffolds Mechanical Properties

The mechanical properties of 3D-printed scaffolds are a crucial factor in their successful use in bone tissue engineering and regenerative medicine. The scaffolds should provide adequate mechanical support to promote cell growth and tissue regeneration [95]. However, low mechanical strength is a major challenge in porous scaffolds, limiting their use in load-bearing applications. Therefore, various approaches have been studied to improve the mechanical properties of 3D-printed scaffolds, such as optimized post-processing, compositional modifications, and bioactive liquid phase sintering aids [96,97]. Additionally, monomer or polymer infiltration has been shown to increase the strength of ceramic scaffolds without impairing their biological properties [98]. An in vivo study demonstrated that the 3D-printed polycaprolactone/β-tricalcium phosphate scaffold with PRP/gelatin microspheres led to greater positive effects in promoting large bone defect repair [99]. Bose et al. [100] provided an in-depth analysis of the mechanical properties of 3D-printed ceramic scaffolds and proposes various approaches to enhance their mechanical strength, such as optimized post-processing, compositional modifications, bioactive liquid phase sintering aids and the use of microwave sintering, as well as the use of monomer or polymer infiltration to improve strength without compromising the biological properties of the scaffolds. Adel-Khattab et al. [101] generated a tissue-engineered synthetic bone graft in vitro that mimicked the beneficial qualities of autogenous bone grafts by having homogenously distributed terminally differentiated osteoblasts and mineralizing bone matrix. As a result, the tissue-engineered synthetic bone graft was successful in being produced and displayed superior mechanical and biological properties. Xu et al. [102] conducted a study on 3D-printed scaffolds made of polycaprolactone (PCL). Their mechanical test results revealed that the compressive modulus increased with a decrease in PCL porosity. In particular, the calvaria-like PCL scaffold with inhomogeneous porosity (average of 35%) exhibited better mechanical properties compared to the PCL scaffold with homogeneous porosity of 35%.

Barak et al. [103] provided compelling evidence of the impact of bone resorption on the mechanical properties of trabecular bone. The decrease in structural strength from an average of 9.14 ± 2.85 MPa to 6.97 ± 2.44 MPa and the decrease in structural stiffness from an average of 282.5 ± 63.4 N/mm to 233.8 ± 51.2 N/mm are substantial and demonstrate the severity of the impact of bone resorption on mechanical properties. They used 3D printing technology in their study to represent a novel and valuable approach for quantifying the effect of structural deterioration on trabecular bone. The findings from their study have the potential to improve fracture risk assessments and personalized treatment plans for patients suffering from excessive bone resorption in the future. Wang et al. [104] presented a promising approach for improving the mechanical stability of decellularized extracellular matrix hydrogel (dECM-G) by combining it with photocross-linkable gelatin methacrylate (GelMA), which retains high bioactivity and tissue-specificity, making it a potentially useful material for regenerative medicine applications. Finally, the printing parameters can also have a significant impact on the mechanical properties of the scaffold [105]. For example, increasing the layer thickness or printing speed can lead to a decrease in mechanical strength while decreasing the layer thickness or increasing the printing temperature can improve the mechanical properties [106].

The design of the scaffold can also play a crucial role in determining its mechanical properties. A scaffold with a highly porous structure, such as a honeycomb or lattice design, can provide sufficient mechanical support while also allowing for cell infiltration and nutrient diffusion [107]. Overall, the mechanical properties of 3D-printed scaffolds for bone tissue engineering depend on a variety of factors, including the material used, the printing parameters and the scaffold design. By carefully optimizing these parameters, it is possible to create scaffolds with the ideal mechanical properties for bone tissue engineering application. See Table 4 for a compilation of 3D printing technologies for bone tissue engineering.

## 8. Maintaining Scaffold Porosity by 3D Printing

**Figure 7:**
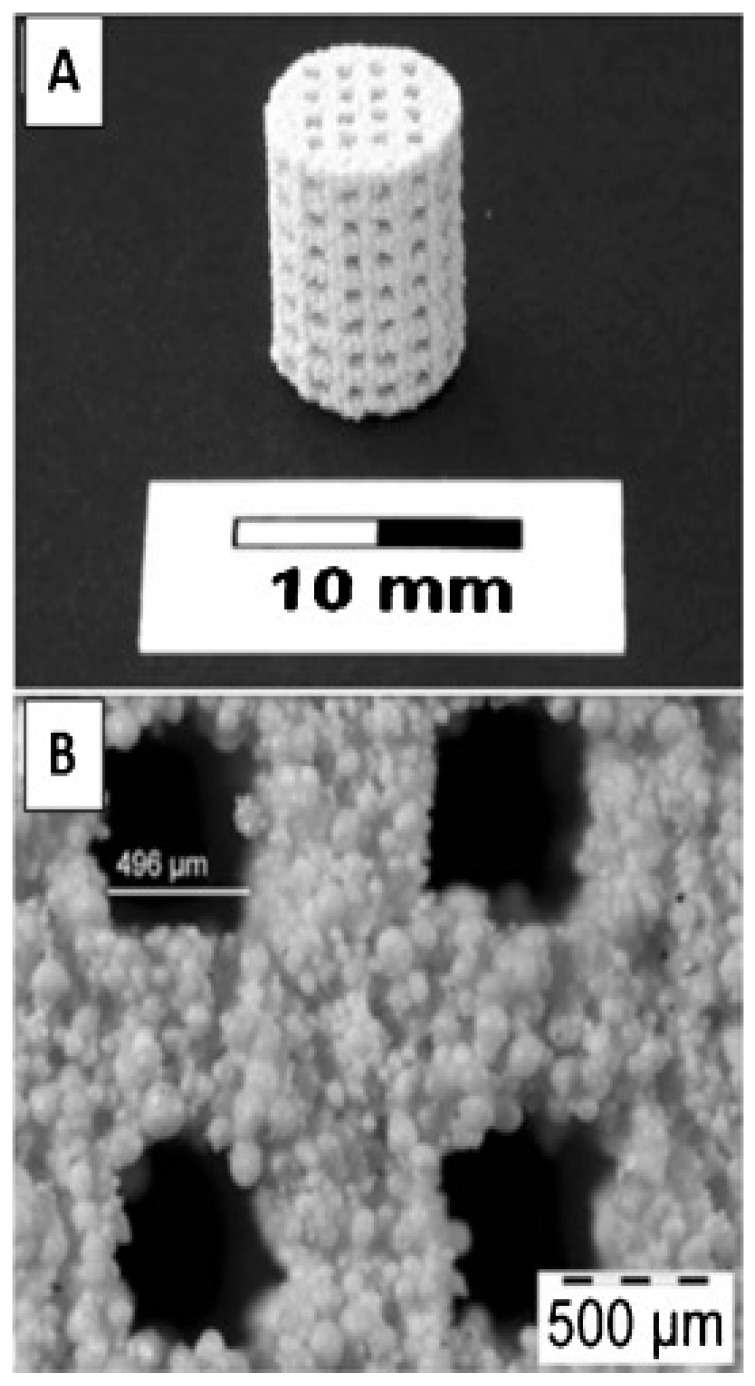
3D-printed hydroxyapatite scaffold with interconnecting channels. (**A**) The macrostructure contains interconnecting channels with (**B**) visible porous structures resulting from polymeric additives [143].

The porosity of the scaffold is essential to provide the necessary space for tissue and cell growth. Figure 7 displays a 3D-printed scaffold that contains interconnecting channels and visible porous structures resulting from polymeric additives. The use of porous scaffolds has become a popular choice as an alternative to traditional scaffolds. The porous structure can facilitate the formation of bone within the scaffold. Bone is highly vascularized, with the majority of these vessels situated within 100 μm of the osteoblasts responsible for synthesizing and mineralizing the bone matrix [129]. Zocca et al. [130] created highly porous (>60% open porosity) glass-ceramic scaffolds with exceptional mechanical qualities (compression strength of 15 MPa). EI-Ghannam et al. [131] established a process that involves crushing the powder at 50 MPa and heating it at 900 °C for two hours. NaOH chemically altered the surface of silicon carbide (SiC) to promote sintering and promote bioactivity. Using 40% PEG, porous discs with 51.51 to 3.17% porosity and interconnected pores between 1 and 1000 m in size were created. Together, the porous SiC scaffolds can be used in trauma surgery as a bone transplant for tissue repair and cell delivery. Yao et al. [132] discussed the ideal synthesis conditions, as well as the kinetics of calcium phosphate (Ca-P) phase development at the surface of PLGA/bioactive glass (BG) composites. The efficacy of PLGA-30%BG-microsphere-based porous scaffolds for bone tissue engineering to encourage osteogenesis of marrow stromal cells (MSC) was investigated. Jones et al. [133] developed bioactive glasses scaffold made from sol-gel materials, which meet the requirements for the perfect scaffold for bone tissue engineering. Since scaffold design progresses, multiple factors should be considered when determining porosity. Basic variables such as the percentage of porosity determined through Equation (1) [134] and pore size are still used, yet they are now complemented by more precise factors such as the complexity of the channels’ shape and the surface area to volume ratio [135]. If the porosity of a scaffold is not designed correctly, it can lead to negative effects such as tissue death, prolonged healing times and increased risk of infection [136]. On the other hand, a scaffold with a properly designed three-dimensional porous structure and an appropriate level of stiffness can promote successful healing by minimizing stress shielding and positively impacting the process of bone integration and healing [137]. The optimal design of the pores within the scaffold is crucial for a favorable biological response. The effect of the porosity in scaffolds on cell activity is not completely understood, so there has been disagreement between studies over the ideal size of the pores to use for bone regeneration. Various pore sizes from 20–1500 μm have been utilized in bone tissue engineering [64,138,139]. In the non-biodegradable material scaffolds, it is recommended that the pore size be larger than 100 μm for cell attachment and larger than 300 μm for tissue growth [140]. However, biodegradable material scaffolds with smaller pore sizes can work perfectly for biological response [141,142]. Over time, there have been many conventional techniques that have been used to modify and control the porous structure of scaffolds. Some of the disadvantages of using these conventional methods include their relatively high costs, the potential for damage to the scaffold structure and the length of time required to complete the process. Additionally, the processes may not be suitable for all types of scaffold materials, and the resulting porosity may not be uniform throughout the scaffold.

Recently, these disadvantages are being overcome by using 3D printing techniques for making high porous scaffolds with the added ability to create complex and highly customizable geometries, precise control over pore size and distribution and the ability to use a variety of materials. A computer-designed scaffold refers to a scaffold that is designed and fabricated using computer-aided design (CAD) software and 3D printing technologies. This allows for precise control over the scaffold’s shape, size and porosity. Additionally, 3D printing techniques can be relatively cost-effective and efficient compared to traditional manufacturing methods. The accuracy of 3D printing technologies is very high, so we do not need to separately calculate the pore size of the scaffold material like before researchers did by thermoporometry (TPM) [144]. The accuracy of 3D printing products can be easily measured by using various techniques such as coordinate measuring machine (CMM) [145], optical comparator [146] and 3D scanning [147]. These techniques can be used to measure the dimensional accuracy, surface finish and overall quality of the printed product. Additionally, software tools such as STL files can be used to compare the design file to the printed product to check for any discrepancies or errors of the printing products. Quality checking regarding the morphological structure for biological properties such as cell viability and biocompatibility can be done through various techniques such as transmission electron microscopy (TEM) [148], scanning electron microscopy (SEM) [149], X-ray diffraction (XRD) [150] and Fourier-transform infrared spectroscopy (FTIR) [151].

(1) $$Porosity\left(\%\right)=\frac{W_{f}-W_{i}}{d_{ethanol}\times V_{scaffold}}$$

where *W_f_* and *W_i_* represent the final and initial scaffold weight, respectively, *d_ethanol_* is the ethanol solution density and *V_scaffold_* represents the volume of the scaffold.

## 9. Scaffold Structural Requirements

Vascularization remains a problem for successful fusion of larger grafts (order of mm in dimensions) in vivo. Implanted bone grafts depend on blood vessels for nutrients, oxygen and growth factors during the initial stages of fusion. The three-dimensional architecture (pore size, porosity and inter-connectivity of pores) of the scaffold used should facilitate spontaneous vascular ingrowth; failing to do so may lead to cell death due to hypoxia in the interior portions of the graft [152]. The necessity of graft implantation occurs primarily to treat large sized defects (order of mm). Hence, it is implied that the microstructural properties of scaffolds should resemble that of cancellous bone also. From Table 5, it can be observed that very few synthetic-polymer-based fabrication techniques provide the necessary pore sizes.

However, the porosity values are sufficiently higher, which may cause more uniform degradation in vivo. In terms of mechanical strength, it will be necessary to rely on other support structures to withstand the compressive/tensile stresses at the defect site. Loads within a human intra vertebral disc can reach a maximum of 4 MPa [108]. The Young’s modulus of a human cortical bone is ~35 GPa, and that of cancellous bone varies between 50–500 MPa [165]. However, the % porosity and mean pore size of most synthetic polymer scaffolds are comparable to that of a cancellous bone (90% [166,167] and ~50 μm) [168]. Using these scaffolds in conjunction with calcium phosphate cements (CPC) may be a feasible solution [9]. Though biologically inert [11], CPC is extensively used to harden materials/fillers to treat minor skeletal defects [169].

## 10. Current Bone Tissue Engineering Scaffold Models

In recent years, extensive research has been done on scaffold assisted bone regeneration using synthetic materials [170]. Using BMSCs (bone marrow stem cells) or osteoblasts on synthetic materials on mammals (mice, rats, and rabbits) is the common model of an in vitro study [122,171]. In 2004, Lendenckel et al. successfully treated and healed a calvarial defect of a seven-year-old girl using hASCs due to a lack of enough autologous cancellous bone from the iliac crest [172]. Still, ethical reasons prevent testing the scope of hASCs combined with a synthetic material on treating skeletal defects in human species [173].

**Synthetic Polymer Models:** On-site delivery of bone marrow stem cells (BMSCs) using a porous polyethylene glycol–polyurethane (PEG–PU) scaffold to the injury site bearing the therapeutic potency of BMSCs. Though no differentiation pathways were performed, this study re-emphasizes the retention of stem cells characteristics in vivo by measuring Oct-4, Sox-2, Klf-4 and c-Myc along with nestin, CD49f, CD29, CD73, CD44 and Sca-1 gene expressions [111,112]. PCL scaffolds were manufactured by the electrostatic fiber spinning method by making a polymer solution of PCL in chloroform. Mesenchymal stem cells (MSCs) derived from bone marrow of neonatal rats (3–7 days old) were loaded on the scaffold (pre-soaked in purified collagen) by mechanically pressing the cell pellet on the scaffold. The electrospun PCL scaffolds exhibited a mineralized bone tissue formation and three-dimensional cell penetration. The shapeability of such tissue-engineered bone grafts in surgical applications for treating defect sites was emphasized [36].

Three-dimensional 75:25 PLGA scaffolds were fabricated by the solvent casting particulate leeching method [75]. Specifically, they were 12.5 mm in diameter and 6 mm in height with a porosity of 79%. MSCs loaded on top of the scaffolds were cultured in a spinner flask and a rotating wall vessel, in addition to static conditions. This was done to study if mitigated nutrient transfer from the media outside the scaffold boundary influenced cell proliferation and differentiation inside the scaffolds. Interestingly, the spinner flask showed comparatively higher cell proliferation and significantly high calcium deposition (representing a high expression of ALP and OC genes) compared to a static culture. On the other hand, rotating wall vessel setup exhibited relatively lower cell proliferation and differentiation. However, in all three culture systems, inhomogeneous cell distribution was observed, with a high cellular density on the surface and a considerably lower density on the interior. This indicated that the microstructural properties of the scaffold itself could determine the three-dimensional cellular growth inside the scaffolds [114].

In situ forming scaffolds eliminate the complexity of fabricating complex geometries ex vivo. Poly(methyl methacrylate) (PMMA) bone cements are the most widely used injectable and in situ forming materials in orthopedics. Hydrogel disks (10 mm diameter and 1 mm thick before swelling) were fabricated with 10 wt% PEGDA in phosphate buffered saline (PBS) with the addition of no Acr-PEG-RGD, 0.5 mM Acr-PEG-RGD, 5.0 mM Acr-PEG-RGD and 5.0 mM Acr-PEG-RDG. Osteoblasts were seeded onto sterile disks at a density of 5 × 10^4^ cells/cm^2^ [115]. Commercially available PLGA scaffolds (GC corporation, Itabashi-ku, Tokyo, Japan) that were 5 mm in diameter and 1.5 mm thick were used to heal large cartilage defects in rabbit knees. These scaffolds were loaded with BMSCs obtained from isolated from the humeral head of each rabbit and cultured in vitro overnight before surgically implanting into the defect areas of the same rabbits. After 4 and 12 weeks of implantation, the defect sites were histologically analyzed. It was found that the implant had fused successfully into the host bone providing architectural support. However, in vivo chondrogenesis was hinted without a dedicated study for gene markers [116].

Membranes made from nanofibers of PLGA was fabricated by electrospinning technique by dissolving PLGA in N,N-dimethylformamide (Junsei, Tokyo, Japan) and tetrahydrofuran (Junsei) solution at 25% (wt). The lactic acid/glycolic acid content ratios were 75:25, 50:50 and a blend of 75:25 and 50:50. Chondrocytes isolated from porcine articular cartilages were cultured on these polymer membranes to study their cellular response in terms of cell proliferation and cytotoxicity. ECM formation (indicative of chondrogenesis) was evaluated by measuring glycosaminoglycans (GAG) content. The chondrocytic phenotype was aspired to be maintained by means of mechanical stimulation as intermittent hydrostatic pressure (IHP). The mechanical strength (tensile modulus, ultimate tensile stress/strain) of these nanofiber-based scaffolds was comparable with human skin and lower than cartilage. Faster degradation of 50:50 PLGA scaffolds was observed, which may be attributed to the hydrophilic nature of glycolic acid content [121].

A custom hybrid poly-(lactic-co-glycolic acid) (PLGA)–gelatin/chondroitin/hyaluronate (PLGA–GCH) scaffold was developed to evaluate its chondrogenic potential relative to PLGA scaffolds. PLGA scaffolds were developed by the low-temperature deposition method (LDM) [41]. The idea was to hybridize PLGA scaffolds with GCH miming the hyaline cartilage ECM composition (15–20% collagen type II, 5–10% chondroitin sulfate, 0.05–0.25% hyaluronan). Cell proliferation and ECM formation were significantly higher in PLGA-GCH hybrid scaffolds than the control PLGA at 24 days in vitro. At 24 weeks post-operation, the hyaline repair was more tenacious and ill-demarcated in PLGA–GCH group at the repair interface [122]. PLGA scaffolds were fabricated by the molding and leeching process combined with sieved NH_4_HCO_3_/NaCl (1:1) particulates of two different size ranges (125–180 and 300–500 μm) and chloroform. Three groups of such scaffolds were surface modified with 0.2% (*V*/*V*) pig-skin collagen type I (Life Technologies, Grand Island, NY, USA), 2% (*W*/*V*) chitosan (Mol. Wt.: 880,000; degree of deacetylation: 90%) (Kiotek, Taiwan) and 10% (*W*/*V*) N-succinyl-chitosan solutions. The results showed that collagen increased cell attachment and proliferation while decreasing osteogenic differentiation compared to the chitosan and N-succinyl-chitosan modifications [118].

PLGA scaffolds of 7 mm in diameter and 4 mm in thickness with 90% porosity were prepared by solution-casting/salt-leaching method. The in vitro degradation analysis shows that it lost around 60% of its original mass at 20 days. The cell attachment study shows that only 37% of the initial loaded cells attached to the scaffolds on day 1. After 2 weeks, calcification was observed in vitro for osteogenic induction medium. Critical size osseous defects of 12 × 5 mm were made on the mandible of 12 mature white New Zealand rabbits (2.5 kg). Tissue-engineered PLGA/MSCs composites were implanted into the defects in the experimental group and just the PLGA scaffolds in the control group. After 6 or 12 weeks, all rabbits were euthanized for histological examinations of the healed sites. It was observed that the defects can be completely cured with tissue-engineered PLGA/MSCs graft after 3 months of implantation. On the contrast, blank PLGA showed very little healing [119]. PLGA and PLGA/PVA scaffolds were developed by melt-molding and particulate leeching method. In vitro and In vivo degradation were focused on this study. The hydrophilic addition of PVA fosters the degradation rate of these scaffolds both in vivo and in vitro for the short term. However, during the long term (4 weeks), the total degradation between the test samples of wetted and pre-wetted PLGA and PLGA/PVA were not significant [117].

Bone marrow stem cell knitted PLGA scaffolds were used to treat tendon defects in New Zealand white rabbits with single sutures as control. Histological examinations were conducted after 2, 4, 8 and 12 weeks of implantation. Type I and type III collagen fiber formation was observed in in vitro cultures of BMSCs combined with PLGA and at 4 weeks post-surgery in New Zealand white rabbits [123]. Blended 3D scaffolds of PCL/PLGA were fabricated using solid freeform technology. Compressive strength was determined as the maximum stress (0.8 MPa) from the linearity of stress-strain curve deflects. The compressive stress modulus of such scaffolds was 12.9 MPa, and the porosity was 69.6%. MC3T3-E1 cells were cultured on these scaffolds with standard culture media (DMEM, 10% FBS) for 15 days with consistent cell proliferation [120].

PLGA scaffolds were fabricated by solid-liquid phase separation method for oriented microporous structures or non-oriented micro-porous structures using NaCl particles as porogeny. In the oriented group, pores were parallelly longitudinal in the vertical section and uniformly distributed in the cross section. In the non-oriented group, pores were spherical and non-homogenous in sizes. There was no significant difference between the volume or porosity of scaffolds between the two groups. However, the scaffolds with oriented porous structures show increased compressive modulus (~7 MPa) relative to non-oriented porous samples (~3 MPa). Additionally, oriented porous structures had more homogeneous cellular growth than the non-oriented porous structures in vitro. Thickness and volume shrinkage were observed to be lesser in the oriented group than the non-oriented group fabricated by NaCl porogens. Additionally, oriented groups had more cartilage-specific ECM deposition after 12 weeks of in vivo implantation in nude mice. These crucial results emphasize the importance of homogeneity of pore sizes and structures in tissue-engineered scaffold [113].

Scaffolds of dual-sized pore structures were fabricated by incorporating montmorillonite (MMT) (a family of phyllosilicates (2:1) that comprise of two tetrahedral silica thin layers with a central octahedral sheet of magnesia) into a PLLA solution. To obtain the final scaffolds, electrospinning and salt leaching/gas-foaming methods were adopted in this study. It was observed that the degradation of scaffolds in terms of weight loss and the molecular weight decrease of PLLA was enhanced by the presence of MMT over a course of 40 days. In particular, the molecular weight underwent a sharp decrease from 110,000 to 10,000 from 5 to 15 days. However, there were no significant differences between pure PLLA scaffolds and MMT-infused nanocomposite models regarding decreases in molecular weight over a period of 40 days. Significant differences in weight loss were observed with PLLA/MMT nanocomposite scaffolds [174]. PLLA scaffolds were formed by solvent casting and particulate leaching, simultaneously incorporating NaCl particles as porogens. The median pore diameter of such scaffolds was 62.44 mm, and the porosity was 90.4%. Human bone marrow stem cells were cultured on these scaffolds, aiming for a viable clinical application as a treatment for either osteoarthritic cartilage injury or the degenerate inter vertebral disc (IVD). It was found that differentiated BMSCs in combination with SOX-9 transfection would establish tighter chondrocytic phenotype. Additionally, pre-differentiated BMSCs, when cultured on PLLA scaffolds, synthesize and similar matrix molecules as in vivo [108].

Macroporous poly(l-lactic acid) (PLLA) scaffolds were fabricated from a PLLA–dioxane–water ternary system with added polyethylene glycol (PEG)–PLLA using the thermally induced phase separation (TIPS) method. The cloud-point temperatures of various compositions of (PEG)-PLLA were observed to increase with increasing concentrations of PLLA. MC3T3-E1 cells (osteoblast-like cells derived from mouse calvaria) were cultured on these three-dimensional constructs for 28 days. The addition of amphiphilic diblocks helped in the fabrication of interconnected scaffolds without segregation or sedimentation. Such macroporous scaffolds induced high cell proliferation for the culture period studied [109]. PLLA scaffolds of 95.65% porosity were fabricated by the solid-liquid phase separation method, with no controlled cooling profiles before sublimation process. A subset of these scaffolds was immersed/coated with simulated body fluid (SBF) or simulated body fluid with collagen (SBFC). Saos-2 osteoblast-like cells were loaded and cultured on these scaffolds to measure their viability and ALP activity at 8 days. Scaffolds made with apatite coating and composite coating expressed more ALP activity and viability than PLLA [125].

**Polymer-Ceramic Models:** PLLA scaffolds fabricated by the thermally induced phase separation method were hybridized with nanohydroxy apatite (NHAP) or microhydroxy apatite (MHAP) at different compositions of NHAP. Two different quenching temperatures were used before sublimation to study their structural integrity as well as different compositions of solvent (dioxane/water). The compressive modulus of the scaffolds increased, as expected, with increasing NHAP/MHAP concentrations. Howeverm 100% dioxane had the highest compressive modulus (~8.5 MPa) compared to water substitutions in solvent [175].

To mechanically strengthen a nanohydroxyapatite/collagen (nHAC) composite, a novel nano-HA/collagen/poly(lactic acid) (nHAC/PLA) was developed by adding PLA to the mixture. A combination of molding and the solid-liquid phase separation method was adopted at a quench temperature of −20 °C before lyophilization. The compressive modulus of these composite scaffolds was the highest (~45 MPa) when the PLA composition was 10% in solvent. PLA concentrations of 8% and 12% had moduli of 20 MPa and 40 MPa, respectively. The ultimate compressive strengths of 8, 10 and 12% PLA were 1.3, 1.5 and 1.85 MPa, respectively. These scaffolds were loaded with osteoblasts derived from calvaria of neonatal rats before being implanted into 15 mm segmental defects in the right forelimbs of New Zealand adult rabbits (2.5–3 kg). The porous scaffolds mimicking the natural cancellous bone in terms of composition and microstructure were found to be biologically active when implanted in vivo [126].

The mineralization of a shish-kebab structure of hydroxyapatite on poly(e-caprolactone) (PCL) nanofibers followed a NFSK (noncoherent frequency shifting key) pattern by incubating in a calcium-induced cell culture medium and SBF (simulated body fluid). A comparative tensile strength analysis of aligned and randomly oriented mats was conducted for PCL, NFSK and mineralized NFSK models. The results showed that the randomly oriented structures showed lower ultimate strength for all models. Aligned mineralized NFSK had the highest Young’s modulus (22.5 MPa), followed by aligned NFSK (10 MPa) and aligned PCL nanofibers (2.5 MPa). Fibroblast (L-929) cells were cultured on these pre-mineralized structures to characterize their cytotoxicity and morphological changes. NFSK scaffolds showed similar cytocompatibility to PCL nanofibers [124]. A porous construct was developed by the high internal phase emulsion (HIPE) polymerization technique. Pore surfaces were coated with hydroxyapatite for enhanced biocompatibility. Rat osteoblasts were seeded and cultured for up to 35 days to measure the cell proliferation and formation of bone nodules (extracellular matrix with related minerals), which characterizes functionally mature osteoblasts [127].

## 11. Freezing Effects on Ceramic and Synthetic Polymers

**Figure 8:**
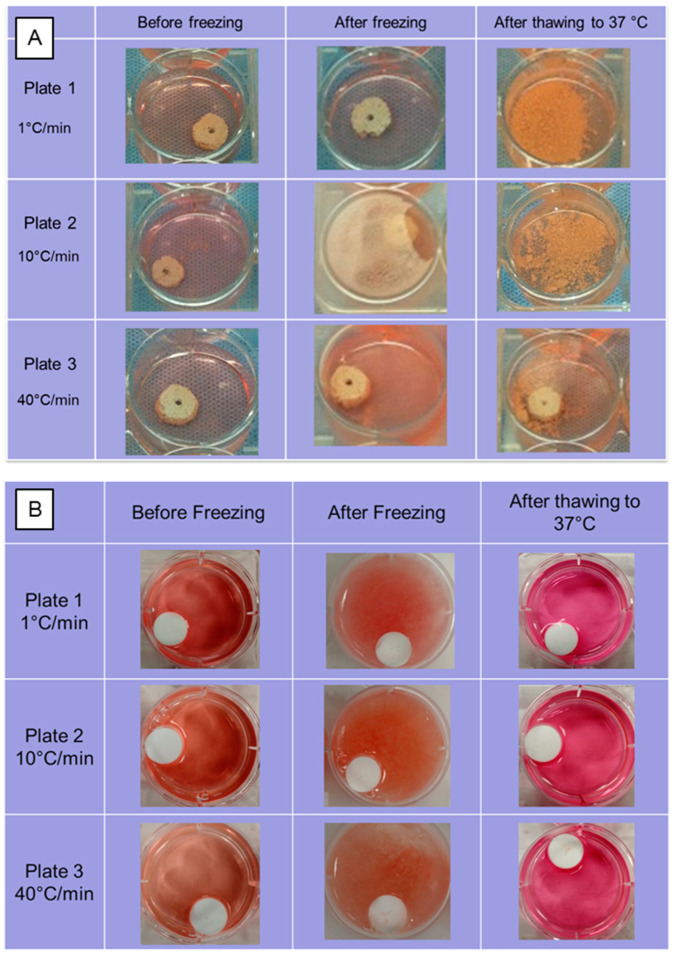
Structural integrity of (**A**) Hydroxyapatite and (**B**) Poly(l-lactic acid) scaffolds when subjected to thermal strains experienced during controlled cryo-preservation process at cooling rates of 1, 10 and 40 °C/min.

Experiments mimicking thermally controlled cryopreservation were run on hydroxyapatite scaffolds made by thermal extrusion process and on PLLA scaffolds prepared by the thermally induced phase separation method using a controlled cooling rate of 10 °C/min pre-lyophilization on 10% (*wt*/*v*) PLLA/dioxane solutions. The scaffolds were cooled to a low temperature of −75 °C at cooling rates of 1, 10 and 40 °C/min using a control rate freezer (CRF) (Planer PLC Group, Shepperton Surrey TW16 7HD, UK) in a growth media. The frozen scaffolds were then stored in liquid nitrogen −196 °C) in MVE CryoSystem 2000 (Chart Industries, Inc., Ball Ground, GA, USA) for 24 h and thawed back to incubation temperature of 37 °C using a water bath. There were several structural changes in the scaffolds due to the thermal strain during the cryopreservation process at various cooling rates, as shown in Figure 8. The ceramic scaffolds completely disintegrated when cooled at 1 and 10 °C/min, whereas partial disintegration was observed when they were cooled at 40 °C/min. Most of the structural damage was visually observed during the thawing process, as the frozen growth media could have kept the structures stable during cooling process. Scaffolds made from PLLA, a thermoplastic polymer, maintained their structural integrity at all cooling rates and low storage temperatures.

## 12. Regulatory Issues

The medical/clinical industry encounters numerous regulatory hurdles when it comes to engineering 3D bone regeneration scaffolds. See, for example, the Federal Drug Administration (FDA) guidelines on human cells or tissues intended for implantation, transplantation, infusion or transfer into a human recipient. These cells or tissues are regulated as a human cell-, tissue-and-cellular- and tissue-based product or HCT/P [176]. Specifically, FDA has issued guidelines on compliance and inspection, donor testing and the registration of tissue banks (to be registered under Human Cell and Tissue Establishment Registration Database) [177]. In the US, the Center for Biologics Evaluation and Research (CBER) regulates HCT/Ps, while the Health Resources Services Administration oversees the transplantation of vascularized human organs. CBER requires tissue establishments to screen and test donors, to prepare and follow written procedures for the prevention of the spread of communicable disease and to establish and maintain good practices and records. In addition, FDA continuously reviews and establishes rules to fully define the scope and range of HCT/Ps and the institutions that are required to comply with the guidelines and regulations. Some of the main challenges besides establishing the proper regulatory classification, conducting wide-ranging preclinical testing to assess safety/efficacy and navigating the intricate process of clinical trials to validate success in humans include maintaining detailed regulatory paper work, creating robust management systems and strategies to assess and handle risks that are inherent to scaffolds’ design and manufacturing, maintaining good manufacturing practices, ensuring appropriate sterilization and packaging for shipping and transport, executing post-market surveillance to monitor performance and safety and addressing different national international regulations when seeking global market access. For example, the European Union has separate and distinct guidelines that are different from the US FDA regulations [178]. Adhering to these regulatory requirements is crucial for ensuring the safety, effectiveness and advancement of 3D bone regeneration scaffolds in clinical applications. A representative compilation of scaffolds for tissue engineering applications is shown in Table 6.

## 13. Conclusions and Future Perspectives

Traditionally, bone implants made from ceramic materials have been implanted into patients. However, in recent years, polymers such as poly(L-lactic acid) and poly(glycolic acid) have been studied extensively for scaffold fabrication for bone grafts. This is mainly due to their high biocompatibility and simple degradation by hydrolysis after implantation. However, for future applications intending to make cryo-preservable bone grafts, pure synthetic polymers may be the only available option. Every aspect of the scaffold fabrication, from the choice of material to the technique, will impact the characteristics of the final scaffolds. Currently, most of the clinical studies involving osteo-chondrogenic models overlook the aspect of mechanical stability. Ultimate strength and compressive/tensile modulus are vital factors when engineering a skeletal graft. The solvent-casting and particulate leaching technique is a simple operation that offers the ability to control pore size and porosity by varying the salt/polymer ratio and particle size of the added salt. Using the gas-foaming technique, scaffolds of high porosity and pore size but low connectivity can be fabricated. With the electrospinning method, large, porous, interconnected scaffolds can be fabricated, but their thickness is limited to microns. Three-dimensional printing gives localized control over the microstructure of the scaffolds. Ultimately, the tissue of interest dictates the characteristics required of a scaffold. For a bone graft, mechanical strength and low cytotoxicity are indispensable. Three-dimensional printing has emerged as a powerful tool in bone tissue engineering, allowing for the creation of scaffolds with precise designs and controlled mechanical properties. The success of these scaffolds depends on the careful selection of materials, printing parameters and scaffold designs. The use of biocompatible materials, combined with optimal printing parameters and designs such as highly porous honeycomb or lattice structures, can produce scaffolds with ideal mechanical properties for bone tissue engineering. As this technology continues to evolve, 3D printing is poised to revolutionize the field of bone tissue engineering, offering new solutions for bone repair and regeneration. The thermally induced phase separation method could be a suitable fabrication method as the low temperature cooling process increases the mechanical strength. At the time of publication, more clinical studies are needed to further evaluate the potential of polymer-ceramic models for viable cryo-preservable bone grafts.

## Figures and Tables

**Table 1:** Biodegradation and mechanical properties of synthetic polymers [30].

Polymer	Biodegradation Time (Months)	Compressive * or Tensile Strength (MPa)	Modulus (GPa)
**PDLLA**	12–16	Pellet: 35–150 * Film or disk: 29–35	Film or disk: 1.9–2.4
**PLLA**	>24	Pellet: 40–120 * Film or disk: 28–50 Fiber: 870–2300	Film or disk: 1.2–3.0 Fiber: 10–16
**PGA**	6–12	Fiber: 340–920	Fiber: 7–14
**PLGA**	Adjustable: 1–12	41.4–55.2	1.4–2.8
**PCL**	>24	-	-

The * is to distinguish between compressive and tensile strengths reported in the table.

**Table 2:** Comparison of Various 3D Printing Processes.

Techniques	Working Principle	Cartilage Scaffold Materials	Biocompatible	Biodegradable	Merits	Demerits
FDM	Heating, melting and Extrusion	Poly(ε-caprolactone)/poly(3-hydroxybutyrate-co−3-hydroxyvalerate) (PCL/PHBV) [46]	Yes [61]	Yes [61]	Inexpensive yet durable, and able to be used with multiple materials	Low Resolution, Anisotropy, clogging nozzle
		Thermoplastic (Nylon, ABS, TPU, PLA, PET, etc.)	No	No		
SLA Laser Based	Laser beam to scan and harden a UV-sensitive material	Hydrogel Based on poly(ethylene glycol)/poly(d,l-lactide) [56], poly(ethylene glycol) (PEG) [62]	Yes [56]	Yes [56]	Printing accuracy and quality are very good for complex scaffold structure	Lifespan of laser is short, High cost
SLA DLP Based	2D images of a 3D model are projected and UV induced curing of these images	Silk fibroin with glycidyl-methacrylate(Silk-GMA) [49],poly (ε-caprolactone) diacrylate/poly (ethylene glycol) diacrylate/chitosan [63]	Yes [49]	Yes [49]	Faster than Laser based SLA	Limitation of object size
SLS	Using laser technology and applying heat to solidify	Polycaprolactone [64]	No	No	High durability, simple to clear away support material	High cost of equipment and materials, deformation of the printed part,
		Titanium [65]	Yes [65]	No [66]		
LAB	Laser beam is directed through a mask or a digital micromirror device to pattern the bioink	Collagen and nano-hydroxyapatite [67]	No	No	High printing resolution	Expensive, Complex, less commercial use
3DP or Inkjet	Droplet-based 3D printing that uses thermal or piezoelectric forces to eject droplets	Acrylated poly(ethylene glycol) (PEG) hydrogel [68]	Yes	No	Excellent microstructural and mechanical properties	Nozzle clogging, nonuniform droplet size
3D Bioprinting	Pressurized syringe extrusion combined with UV light to set the material in place.	Gelatin methacrylate, polycaprolactone, hyaluronic acid, polylactic acid	Yes		High degree of customization	Difficult to achieve uniform microarchitecture

**Table 3:** Summary of composite sintering approaches outlining the specific print parameters utilized, physical attributes and biological outcomes of the printed constructs. Where P = Laser Power, λ = Wavelength, S = Scan Spacing, T = Layer thickness, V = Scan Velocity, Φ = Beam Diameter, E = Elastic Modulus, σUC = Ultimate Compressive Strength [79].

Composite Formulation(s)	Print Specifications	Physical Attributes	Biological Response	Ref.
PCL/HA In wt% ratios of 100:0, 90:10, 80:20 and 70:30	P = 1–1.2 W λ = 10.6 µm S = 152.4 µm T = N/A V = 914 mm/s Φ = 450 µm 50 °C bed temp	Increased HA concentration resulted in a higher E but a reduction in σUC		[80]
PCL/β-TCP In wt% ratios of 100:0, 90:10, 50:50, NB 50:50 utilised smaller PCL particles	P = 7 W λ = 10.6 µm S = N/A T =0.11 mm V = N/A Φ = 410 µm 49 °C bed temp	Increasing β-TCP content was found to decrease the strength		[81]
PLLA/GO@Si-HA	P = 7 W λ = N/A S = N/A T = N/A V = 180 mm/s	Compressive strength and modulus improved by 85% and 120% after incorporating GO@Si-HA, with a marginal improvement in hardness	4 wk SBF: PLLA minimal, PLLA/GO minimal, PLLA/GO@Si-HA significantly improved appetite formation and MG-63 cell morphology and ALP activity after 7 days	[82]
PEEK PEEK/20%plyglycolicacid (PGA) PEEK/40%PGA	P = 100 W (max) λ = 10.6 µm S = 2.5 mm T =0.1–0.2 mm V = 400 mm/min Φ = 800 µm	Increase in PGA concentration reduced compressive and tensile strength	PGA had no significant influence on MG-63 cell viability or morphology	[83]
Poly (vinylidene fluoride)/Bioactive glass 58s (PVDF/58s)	P = 100 W (max) λ = 10.6 µm S = 3 mm T = 0.1–0.2 mm V = 500 mm/s Φ = 800 µm	BG was found to be slightly exposed on the surface of scaffolds following EDS analysis	BG 58s addition improved osteoconductivity and osteoinductivity of scaffolds, following SBF and MG-63 cell seeding analysis	[84]
Aliphaticpolycarbonate/HA(aPC/HA) a-PC a-PC/5 wt% HA a-PC/10 wt% HA a-PC/15 wt% HA	P = 11 W λ = 10.6 µm S = 0.15 mm T =0.15 mm V = 2000 mm/s Φ = 200 µm 135 °C bed temp	Surface roughness and porosity (53 to 82%) increased with HA content, below 15 wt% ideal 6–7 times reduction in scaffold strength with HA compared to pure a-PC	Osteoconductivity unchanged by SLS processing	[85]
Poly [3,6-dimethyl-1,4- dioxane-2,5- dione]/HA	P = 10 W λ = 1.06 µm S = N/A T = N/A V = mm/s Φ = 125 µm	Young’s modulus increased from 6.4 to 8.4 GPa with HA addition	Sintered composite scaffolds improved ATSC attachment and viability, compared to foaming method and virgin polymer	[86]
PVA/HA 90:10 vol% 10–75 µm 50–100 µm	P = 10–20 W λ = 10.6 µm S = N/A T = N/A V = 1270–2540 mm/s and 2032 mm/s 65–75 °C bed temp and 80 °C bed temp for larger particles	Ball mixing was found to be best for homogenous blends of PVA and HA when compared to tumbler mixer. Larger particles also prevented clumping during layer deposition		[50]
PCL PCL/TCP PCL/TCP/collagen	P = 1 W (PCL) and 2 W λ = N/A S = 0.2 mm T = N/A V = 500 mm/s 49 °C bed temp	Significant improvement of compressive modulus with addition of TCP, col no difference	Improved pASC attachment, viability and osteogenic differentiation (ALP and osteocalcin) with TCP and TCP/col addition, ALP activity highest at day 7 for all scaffolds (over 28 days). Woven bone and vasculature observed in vivo with composites, pure PCL was full of fibroblasts and granular tissue	[87]

**Table 4:** Current Osteo-Chondrogenic models.

	Scaffold Material	Cell Type	Species	Cell Loading Density	Study Type	Pathways	Gene Markers	Period of Culture	References
**Polymers**	PLLA	BMSCs	Male human	5 × 10^5^/scaffold	In vitro	Chondrogenesis	SOX-9, COL1A1, COL2A1, Aggrecan	4 weeks	[108]
	(PEG)–PLLA	MC3T3-E1 cells	Mouse calvaria	6 × 10^5^/scaffold	In vitro	--	--	4 weeks	[109]
	mPEG-PCL gel	hADSCs	Fisher rat	2.5 × 10^5^/scaffold	In vitro/In vivo	OS	ALP	3 weeks (In vitro), 4 weeks (In vivo)	[110]
	polyethylene glycol–polyurethane (PEG–PU)	BMSC	C57BL/J6 mice	5 × 10^5^/scaffold	In vitro/In vivo	Engraftment	Sca-1, CD11b, CD29, CD133 and CD140a	10 days	[111,112]
	PLGA	Chondrocytes	Newborn swine	5 × 10^7^/cm^3^	In vitro/In vivo	--	--	12 w (In vitro), 12 w (In vivo)	[113]
	PCL	BMSCs	Lewis rats	4 × 10^6^/scaffold	In vitro	OS	ECM, Calcification	4 weeks	[36]
	75:25 PLGA	MSCs	male Sprague–Dawley rats	10^6^/scaffold	In vitro	OS	ALP, OC	21 days	[114]
	Poly(ethylene glycol)-diacrylate (PEGDA)	Calvarial Osteoblasts	Rats	5 × 10^4^/cm^2^	In vitro	OS	--	4 weeks	[115]
	PLGA	BMSCs	Male Japanese white rabbits (3–4 kg)	1 × 10^7^ cells/cm^3^	In vitro/In vivo	Chondrogenesis	--	12 h (In vitro), 12 w (In vivo)	[116]
	PLGA/PVA	--	Male Sprague-Dawley rats (200–250 g)	--	In vivo	--	--	4 weeks	[117]
	Modified PLGA	Osteoblastic stromal cells	Sprague-Dawley rats	7 × 10^4^/scaffold	In vitro	OS	ALP, Ca deposition	14 days	[118]
	PLGA	MSCs	White New Zealand rabbit	10^5^/scaffold	In vitro/In vivo	OS	Ca deposition	20 d (In vitro), 12 w (In vivo)	[119]
	PCL/PLGA	MSCs (MC3T3-E1)	Mouse	--	In vitro	--	--	15 days	[120]
	PLGA	Chondrocytes	porcine	5 × 10^4^/scaffold	In vitro	--	ECM	14 days	[121]
	PLGA–GCH	BMSCs	Mature New Zealand white rabbits (2.5–3 kg)	10^7^/scaffold	In vitro/In vivo	Chondrogenesis	ECM	8 h (In vitro), 24 w (In vivo)	[122]
	PLGA	BMSCs	Female New Zealand white rabbits	1 × 10^7^/graft	In vivo	Tenogenesis	--	12 weeks	[123]
**Polymer–Ceramics**	Poly(caprolactone) (PCL) (nanofibers), hydroxyapatite (HAP)	L-929 fibroblast cells	Mouse	5 × 10^3^/scaffold	In vitro	OS	ALP	5 days	[124]
	PLLA/Apatite/Collagen	Saos-2	Female Human	1 × 10^5^/scaffold	In vitro	--	ALP	8 days	[125]
	nano-HA/collagen/PLA	Osteoblasts	Rat calvaria	5 × 10^4^/cm^2^	In vitro/In vivo	--	--	16 weeks	[126]
	PolyHIPE Polymer	osteoblast cells	Rat	300 × 10^5^/scaffold	In vitro	OS	--	35 days	[127]
	poly-ε-caprolactone (PCL)/CaP crystals	BMSCs	Human	3 × 10^5^/scaffold	In vitro	OS	Ca deposition. OC, collagen-I	8 weeks	[128]
	PLGA/HA	Calvarial Osteoblasts	Rat	2.0 × 10^6^/scaffold	In vitro/In vivo	--	Ca deposition	8 weeks	[62]

**Table 5:** Structural properties of scaffolds made from various techniques.

Scaffold Material/Fabrication Method	Pore-Size		Porosity (%)		Compressive Modulus (MPa)	
	Min	Max	Min	Max	Min	Max
**Non-3D-printed ceramic scaffolds [153,154,155]**	300 μm	1 mm	25	80	3	50
**Polymers/Gas foaming [156,157]**	10 μm	100 μm (439 μm with salt leeching)	67	97	0.15	0.3
**Ceramics/Gas foaming [158,159]**	100 μm	400 μm	46.8	78.4	100	1800
**Polymers/Electro-spinning [160,161]**	0.11 μm (fiber dia)	1.19 μm (fiber dia)	-	-	1.09	20
**Polymers/TIPS [37,162]**	50 μm	100 μm	71	91	0.15	6.2
**Cancellous bone [163]**	300 μm	600 μm	75	85	100	300
**Cortical bone [163,164]**	10 μm	50 μm	5	10	18,000	22,000

**Table 6:** Examples for scaffold of polymer blends [179].

Biomaterial Composition	Fabrication	Cell Type	Application	References
Collagen and fibrinogen scaffolds	Inkjet Printing	Chondrocytes	Cartilage	[180]
Gelatin and fibrinogen scaffolds	Extrusion	hMSCs, hUVECs, hNDFs	Vascular	[181]
Alginate and methacrylated gelatin scaffolds	Extrusion	hUVECs	Cardiac	[182]
Nanofibrillated cellulose and alginate scaffolds	Extrusion	Chondrocytes	Cartilage	[183]
Methacrylated hyaluronam and methacrylated gelatin scaffolds	Extrusion	hAVIC	Cardiac	[184]
Thiol hyaluronic acid, thiol gelatin, dECM, and PEG-based crosslinkers in scaffolds	Extrusion	Multicellular primary cell liver spheroids	Liver	[185]
Gelatin, alginate, EGF, and dermal homogenates scaffolds	Extrusion	Epithelial progenitor cells	Sweat gland	[186]
Alginate, gellan and BioCartilage (micronized human cartilage particles) scaffolds	Co-Extrusion	Chondrocytes	Cartilage	[187]
Cell-laden collagen core and alginate sheet scaffolds	Extrusion	hASCs	Liver	[188]
Heparin sulphate—laminine mimetic peptide amphiphile nanofibre scaffold	Freeze-drying	SH-SY5Y	Neurons	[189]
Nanofibrous PET scaffolds coated with collagen	Electrospinning	Caco-2 (human epithelial cells)	Intestinal epithelium	[190]
Polypyrrole-coated paclitaxel-loaded PCL fibrous scaffold	Electrospinning and membrane surface functionalization	-	Site-specific drug delivery platform with NIR (near-infrared) and pH-triggering for synergetic photothermal chemotherapy	[191]
PCL-collagen radially aligned nanofibre scaffolds	Modified electrospinning	rCCs (Rabbit corneal cells)	Cadaveric corneas and amniotic membranes	[192]
Fibroblast loaded collagen-based construct with PCL mesh	Hybrid extrusion and inkjet process	Keratinocytes	Human skin	[193]
TFG-β1 or gentamicin loaded PCL/collagen nanofibres	Electrospinning	Human dermal fibroblasts	Wound healing	[194]
Devitalized native cartilage with porous PCL scaffolds	Electrospinning	ASCs	Cartilage	[195]
Plasma-treated PLLA: PCL (4:1) nanofibrous scaffolds coated with Matrigel	Electrospinning	hESCs	Auditory nerve	[196]
PLLA, agar, and gelatin scaffolds	Thermally induced phase separation	Chondrocytes	Cartilage	[197]
PLLA- fibronectin mimetic peptide fibrous scaffolds	Electrospinning	Human adult renal stem cells	Renal tubular epithelial lineage	[198]

## Data Availability

No new data were created or analyzed in this study. Data sharing is not applicable to this article.
